# Improving Water Quality Index Prediction Using Regression Learning Models

**DOI:** 10.3390/ijerph192013702

**Published:** 2022-10-21

**Authors:** Jesmeen Mohd Zebaral Hoque, Nor Azlina Ab. Aziz, Salem Alelyani, Mohamed Mohana, Maruf Hosain

**Affiliations:** 1 Faculty of Engineering & Technology, Multimedia University, Melaka 75450, Malaysia; 2 Center for Artificial Intelligence (CAI), King Khalid University, Abha 61421, Saudi Arabia; 3 College of Computer Science, King Khalid University, Abha 61421, Saudi Arabia

**Keywords:** water quality index, regression, linear regression, ridge

## Abstract

Rivers are the main sources of freshwater supply for the world population. However, many economic activities contribute to river water pollution. River water quality can be monitored using various parameters, such as the pH level, dissolved oxygen, total suspended solids, and the chemical properties. Analyzing the trend and pattern of these parameters enables the prediction of the water quality so that proactive measures can be made by relevant authorities to prevent water pollution and predict the effectiveness of water restoration measures. Machine learning regression algorithms can be applied for this purpose. Here, eight machine learning regression techniques, including decision tree regression, linear regression, ridge, Lasso, support vector regression, random forest regression, extra tree regression, and the artificial neural network, are applied for the purpose of water quality index prediction. Historical data from Indian rivers are adopted for this study. The data refer to six water parameters. Twelve other features are then derived from the original six parameters. The performances of the models using different algorithms and sets of features are compared. The derived water quality rating scale features are identified to contribute toward the development of better regression models, while the linear regression and ridge offer the best performance. The best mean square error achieved is 0 and the correlation coefficient is 1.

## 1. Introduction

Surface water, especially that from rivers, is the main source of fresh water and is important for ecology, social well-being, and economic development [1,2]. According to a report by the United Nations Environment Program (UNEP) [3], in some countries, surface water comprises up to 90% of the population’s main drinking water sources. In the same report, the UNEP reported three important findings: (1) that severe pathogen pollution affected one-third of the rivers in Latin America, Africa, and Asia, (2) that severe organic pollution was observed in one-seventh of the rivers of the same continents, and (3) that moderate to severe salinity pollution in one-tenth of the rivers was reported. Therefore, realizing the seriousness of the issue, one of the United Nation’s (UN) 15-year Sustainable Development Goals (SDG) is to ensure water access and sanitation (Goal 6) [4]. This particular goal aims to guarantee the right of the world population, regardless of economic status, to have access to clean drinking water and sanitation. In total, 193 of the UN members signed the pledge to strive for this goal.

The water pollution in rivers is influenced by a variety of causes, including natural factors, such as rainfall and land erosion [5], and human activities, such as urbanization, agriculture, and manufacturing [6]. Developing countries frequently experience rapid economic expansion, and every development initiative has the potential to have negative environmental consequences. Development also attracts population growth, which increases the demands for food production by the agricultural sector. This puts stress on the soil’s natural fertility as a result of the over-extraction of nutrients and necessity for the use of chemical fertilizers. The fertilizers then run into rivers and groundwater, polluting the water sources and wreaking havoc on the ecosystem and human health. Thus, the need for efficient water quality monitoring and assessment is becoming more pressing and critical with respect to human and environmental health protection, and there is also a need for effective, long-term water management.

Many countries have water monitoring systems that monitor the water quality through various water quality parameters. Among the parameters are dissolved oxygen (DO), potential hydrogen (pH), electrical conductivity (CO), biological oxygen demand (BOD), nitrate (NO_3−_), faecal coliform (FC), and total coliform (TC). The water quality parameters are often combined into a single value known as the water quality index (WQI), used to quantify the level of water quality [7]. The WQI has been widely used to assess and categorize the quality of both surface and ground waters [8,9,10,11]. An example of a water quality monitoring authority is the Malaysian Department of the Environment under the Ministry of Environment and Water. The department has 233 monitoring stations nationwide [12]. Meanwhile, the United States Geological Survey (USGS) is in charge of monitoring and collecting the water parameters across the states via more than 13,500 stations [13]. The historical data from these stations are approximated to amount to more than 4.4 million. In 2013, it was reported that there are 870 water monitoring stations in India that are monitored by the Central Pollution Control Board (CPCB) under the Indian Ministry of the Environment, Forests and Climate Changes [14]. However, among the main weaknesses of the existing system is a lack of data management and trend analysis.

Over the last few years, artificial intelligence (AI), particularly machine learning models, have been broadly applied to solve many environmental engineering problems, including river water quality prediction modelling [15,16,17,18]. The regression techniques, such as linear regression (LR), decision tree regression (DT), support vector regression (SVR), random forest regression (RF), extra tree regression (ET), ridge, Lasso, and the artificial neural network (ANN), are popularly selected by researchers for the forecasting and prediction of various problems, such as ozone concentration prediction [19], solar thermal system forecasting [20], indoor temperature forecasting for building temperature control [21], and water quality prediction [22,23,24]. However, the best algorithm must be subject to no free lunch theorem, in which there is no ultimate algorithm that best suits all the types of problems or data [25]. In this research, we investigate the application of these eight algorithms for WQI prediction. These techniques are selected based on their reported high performance, as well as their popularity. We aim to identify the best performer among these algorithms for WQI prediction is as a guideline for future research in this area. Indian water quality data are used in this research. The data are freely and openly available for researchers in Kaggle; hence, future researchers will be able to emulate this work without data accessibility issues. Six water quality parameters and two sets of derivative features from the original features are used as the inputs for the algorithms in order to learn the patterns and predict the WQI. The results show that among the eight regression techniques studied, LR and ridge, using the derived features, are able to achieve the zero mean square error (MSE), the highest correlation coefficient (r), the lowest root mean square error (RMSE), and the lowest mean absolute error (MAE), thus reflecting their excellent prediction accuracy. 

The rest of this paper is organized as follows. Existing works in this area are reviewed in Section 2, and this is followed by the methodology applied in this research in Section 3. The results are presented and discussed in Section 4. Directions for future work and challenges can be found in Section 5. Finally, the work is concluded in Section 6.

## 2. Related Works

In this age and era of AI data, such data can be analyzed autonomously, efficiently, and objectively. Therefore, many studies have been conducted on the application of AI for water quality index predictions. The prediction systems are able to guide authorities in making proactive decisions in order to prevent the degradation of water quality and implement suitable measures in addressing water pollution. 

In [9], Hong Kong’s Lam Tsuen River data are used, and the ET machine learning model is applied to estimate the monthly WQI of the river. The ETR model’s performance is compared with SVR and DT. Ten water quality parameters, including the BOD, chemical oxygen demand (COD), DO, CO, pH, nitrate-nitrogen (NO_3−_N), nitrite-nitrogen (NO_2−_N), phosphate (PO_4_^3−^), temperature (T), and turbidity (TUR), are used to create the prediction models. The prediction test performance achieved a correlation coefficient value of 0.98 and RMSE of 2.99. However, the authors use 10 factors, which is costly, as it requires more sensors in order to obtain these data [26]. The work also ignores other regression models that are available. On the other hand, 16 different data mining algorithms are used for WQI prediction, using the BOD, COD, DO, pH, total solids (TS), FC, PO_4_^3−^, NO_3−_, TUR, and CO, in [27]. The data are from two Talar catchment water quality monitoring stations collected over a six-year period (2012–2018). The data refer to 10 input parameters. However, the findings show that not all the parameters are important for ensuring a good prediction accuracy, and the best parameter combination is algorithm-dependent. The FC is observed as the most important, while the TS is the least important. 

In [28], physicochemical data from 19 wells near a shale gas extraction site are applied. The WQI of groundwater is modelled using ANN techniques. There are seven input parameters of the ANN, including CO, pH, calcium (Ca), magnesium (Mg), phosphate (PO_4−_P), potassium (K), and sulfur (SO_4_^2−^), but the best model is achieved using only five input neurons, including CO, pH, Ca, Mg, and K, SO_4_^2−^. The model achieved an RMSE value of 0.651258 and correlation coefficient value of 0.9984. Similarly, the ANN model is used in another study [29]. The WQI was computed from the COD, BOD, DO, suspended solids (SS), pH, and ammoniacal nitrogen (AN) parameters and obtained a high correlation of 98.78%. Nonetheless, there is still room for improvement. The effect of reducing the number of parameters has been considered, but different ML models have not been evaluated. ANN is popularly chosen for WQI prediction [24], and it is also adopted in [30,31] for Malaysian river predictions. The ANN is used in [30] to predict six water quality parameters using 31 input parameters. The six parameters are important pollution indicators. Meanwhile, two ANN architectures, namely the back propagation NN (BPNN) and radial basis function NN (RBFNN), are studied in [31] to predict the WQI based on standard Malaysian water quality parameters, including the DO, BOD, COD, SS, AN, and pH. The effect of excluding BOD in WQI prediction is also investigated in the study, as this parameter measurement is costly. The findings show that excluding BOD in the prediction does not jeopardize the model’s prediction and, additionally, RBFNN was found to be a good model.

In other research [32], the ANN is used to predict different sets of water quality parameters, including the total nitrogen (TN), ammonium (NH_4_^+^), PO_4_^3−^, and COD. The highest performance is associated with the prediction of PO_4_^3−^, with a correlation coefficient value of 0.98. However, the model only predicts individual water quality parameters, rather than the WQI. ANN models are also used in [33] to predict WQI parameters, where the results for the pH, CO, DO, and TUR are presented. Specifically, two multilayer perceptron (MLP) models are employed in the work. A good performance is observed, suggesting that MLP is able to predict the South African water quality well. Similarly, an ANN is used in [34] to predict the WQI value of the Warta River in Poland, using five selected parameters, including the total dissolved solids (TDS), chloride, total hardness (TH), NO_3−_, and manganese. The model obtained a 0.9792 correlation coefficient value. 

Multi-task learning and deep neural networks are studied in [35] for the purpose of water quality prediction. Four multitask structures are proposed in the work, which employs data from 120 water quality monitoring stations in China. The proposed method is compared with seven other models, and the proposed multi-task, gated, hidden parameter shows a significantly better performance.

The works reviewed are tabulated in Table 1. From the reviewed works, it can be seen that water quality prediction research is an active topic among researchers worldwide. This demonstrates the importance of this topic. The performance of the predictor is influenced by the algorithm used, as well as the input parameters. ANN is observed to be the popular choice among the researchers in this area, and the parameters used are not uniform. Additionally, the parameters used and their number also influence the performance. Therefore, this study examines the possibility of improving water quality index prediction through the choice of the algorithm and parameters.

## 3. Methodology

**Figure 1:**
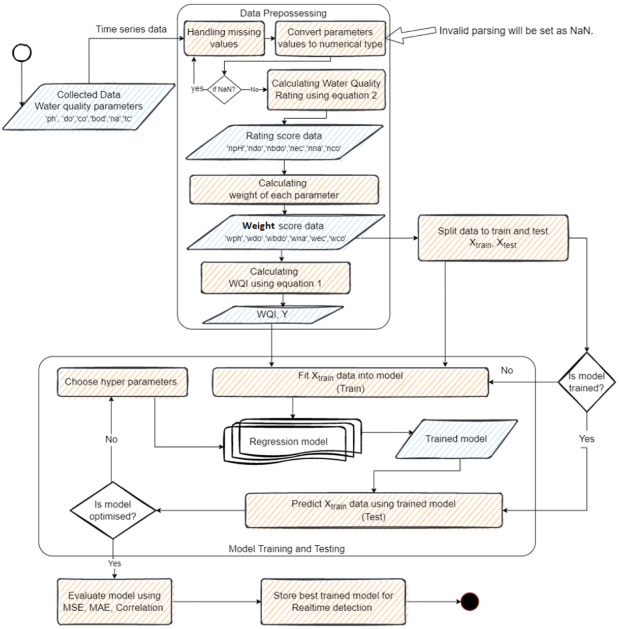
Overall methodology.

The overall structure of the methodology used in this research is illustrated in Figure 1. Indian water quality data are used in this research. They form an open dataset with six water quality parameters. The whole methodology can be broadly categorized into two phases, namely, the data preprocessing phase and regression model training and testing phase.

### 3.1. Dataset

Indian water quality data from Kaggle (https://www.kaggle.com/datasets/anbarivan/indian-water-quality-data, accessed on 1 December 2021) are used in this research. The data are freely available; thus, this work can easily be replicated by using the same dataset. In the dataset, historical water quality parameters from several locations in India are provided. The data were collected between 2003 and 2014, with 1991 samples from various Indian states. These data are used by the Indian government to determine whether the drinking water supplied to the population meets the required standards. 

India has a tropical climate in its southern states, while the northern states have a temperate climate [14]. It has 13 major river basins, including the Brahmaputra, Ganga, Indus, Godavari, Krishna, Mahanadi, Narmada, Cauvery, Brahmini, Tapi, Mahi, Pennar, and Sabarmati. The river basins cover more than 20,000 km^2^ of the surface area. The rivers are mostly perennial and dry up in summer. More than 80% of the rivers are heavily polluted, with the Ganga and Yamuna Rivers being the most polluted [36].

Six water quality parameters from this dataset, including the DO, pH, CO, BOD, NO_3−_, and FC, are used here. These parameters are important for measuring the water quality. DO is among the most important indicators of water quality. Surface water absorbs oxygen due to the aerating effects of winds. A low amount of DO in water may indicate that there are too many bacteria or too much algae present [37]. When the DO level is too low, fish and other aquatic creatures cannot survive [38]. Lower DO is also reflected by higher BOD, which is due to less oxygen being available for oxygen-hungry organisms [39]. Healthy pH levels are also an important water quality indicator. For instance, toxic heavy metals dissolve quickly in acidic water, making the water more harmful to living things [40]. The availability of crucial plant nutrients is similarly affected by the pH, with several nutrients becoming less abundant when the pH is above 7. The next parameter, CO, is a key indication of ionic salt contamination, and it is used to determine the concentration of ionic salts in water. The conductivity of drinking water ranges from 0.05 to 0.5 mS/cm. High CO is not only harmful to health but also destructive to piping infrastructure. Additionally, monitoring the presence of NO_3−_ in water, especially that for domestic usage, is important. Too much nitrate consumption might alter the way in which the blood transports oxygen and lead to methemoglobinemia [41]. Furthermore, nitrate is a good indicator of industrial and urbanization pollution [30]. The last parameter in this dataset is the FC. A high FC reading indicates fecal contamination, with a strong possibility that harmful pathogens, such as Salmonella spp., Shigella spp., Vibrio cholerae, and E. coli, exist in the water supply [42]. Fecal contamination is known to be cause of cholera outbreaks in India [43]. The outbreak had caused loss of many lives.

**Figure 2:**
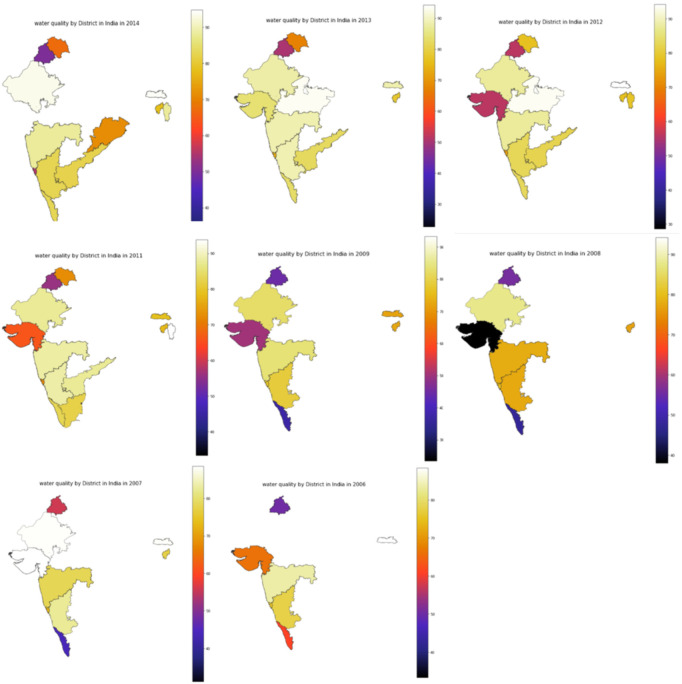
Choropleth map of India’s WQI for 2006–2014.

The water quality index based on this dataset is visualized using choropleth maps in Figure 2 according to the state and selected years. The pre-processing of the locations and state features was performed prior to the plotting of these data. For some of the state data, which are equal to NaN, the state information was obtained from the location feature. However, due to missing data on the state for some of the years, not all the states appear on each map. For example, the data for the Madhya Pradesh state (central India) are only available for 2012 and 2013; therefore, the state only appears in these two years. The lighter color indicates a better water quality, and the darker color indicates a low water quality. The WQI of the dataset ranges from 19.3 to 99.62, according to which the water quality ranges from excellent to poor. The visualization shows that the water quality is not consistent from year to year and varies from one state to another. The water from protected forestlands in the upper catchment is of an excellent quality, has a low level of contamination, and has a very good WQI. 

It is worth noting here that the prediction models used in this research are not year- or location-specific. Hence, the models can be used for any location and time if the same parameters are used.

### 3.2. Data Preprocessing

As a measure used to improve the data quality, data processing is a crucial step in the data analysis process. In this stage, the WQI is calculated using the dataset’s parameters. The WQI is calculated by utilizing the parameters that have a substantial impact on the water quality [26]. The WQI value is calculated using Equation (1) [44].

(1) $$\mathrm{WQI}=\frac{\sum_{i=1}^{n}WQWS_{i}}{\sum_{i=1}^{n}w_{i}}$$

Here, n denotes the number of parameters used to calculate the WQI. The $w_{i}$ represents each feature’s unit weight. Meanwhile, $WQWS_{i}$ is the water quality weight score. It is calculated using Equation (2) [45].

(2) $$WQWS_{i}=w_{i}\times WQR_{i}$$

In Equation (2), $WQR_{i}$ is a value used as a quality rating scale for each feature i and is calculated using Equation (3) [45,46] below:

(3) $$WQR_{i}=100\times \left(\frac{Actual_{i}-Ideal_{i}}{Standard_{i}-Ideal_{i}}\right)$$

where it is calculated using the actual value of parameter i in the tested water samples, $Actual_{i}$, the optimal parameter value i of the pure water$,\;Ideal_{i}$, and the suggested parameter standard value i, the $Standard_{i}$. Table 2 displays the values of $w_{i}$, $Ideal_{i},\;\mathrm{and}\;Standard_{i}$ for each parameter, which can be found in [44,47,48].

In this study, unlike the previous studies, where researchers investigated the combinations, exclusion, and importance of the parameters and their effects on the prediction performance, all six parameters are adopted, and the application of two sets of their derivatives features, namely the $WQWS_{i}$ and $WQR_{i}$, is studied. All 18 features are evaluated as potential inputs. The features are divided into 3 sets. Table 3 shows the three combinations that were created and evaluated. Set 1 (i.e., $qi_{1}$) consists of raw features, including the DO, pH, CO, BOD, NO_3−_ (i.e., Na), and FC. Next, set 2 (i.e., $qi_{2}$) consists of the $WQR_{i}$, calculated using Equation (3). Another set of features (i.e., $qi_{3}$) includes the $WQWS_{i}\;$from Equation (2).

### 3.3. Regression Water Quality Prediction

Previous studies showed that the selection of the learning algorithm influences the quality of the prediction system. Hence, this study used eight standalone regression learning algorithms (DT, LR, Ridge, Lasso, SVR, RF, ET, and ANN) to predict the WQI value.

The standard regression equation serves as the foundation for every type of regression machine learning model and is calculated using Equation (4) [49]:

(4) Y=Xβ+e

where Y is the dependent variable, which, in this case, is the WQI, X stands for the independent variables (i.e., water quality parameters, $qi_{1},qi_{2}$, and $qi_{3}$), β stands for the estimated regression coefficients, and e stands for the errors and residuals.

#### 3.3.1. Decision Tree Regression

The DT model is generated using the provided water quality samples. The DT algorithm, being processed in such a way, is used to identify the optimal tree structure through the minimization of the fitness function. In this work, the DT fitted the output WQI value using each of the independent water quality factors. The dataset is divided into different splitting points of the independent features. The processing of the algorithm generates the error value between the actual and predicted value for each split point. The error is calculated based on the pre-defined fitness functionality. The process continues recursively. 

**Figure 3:**
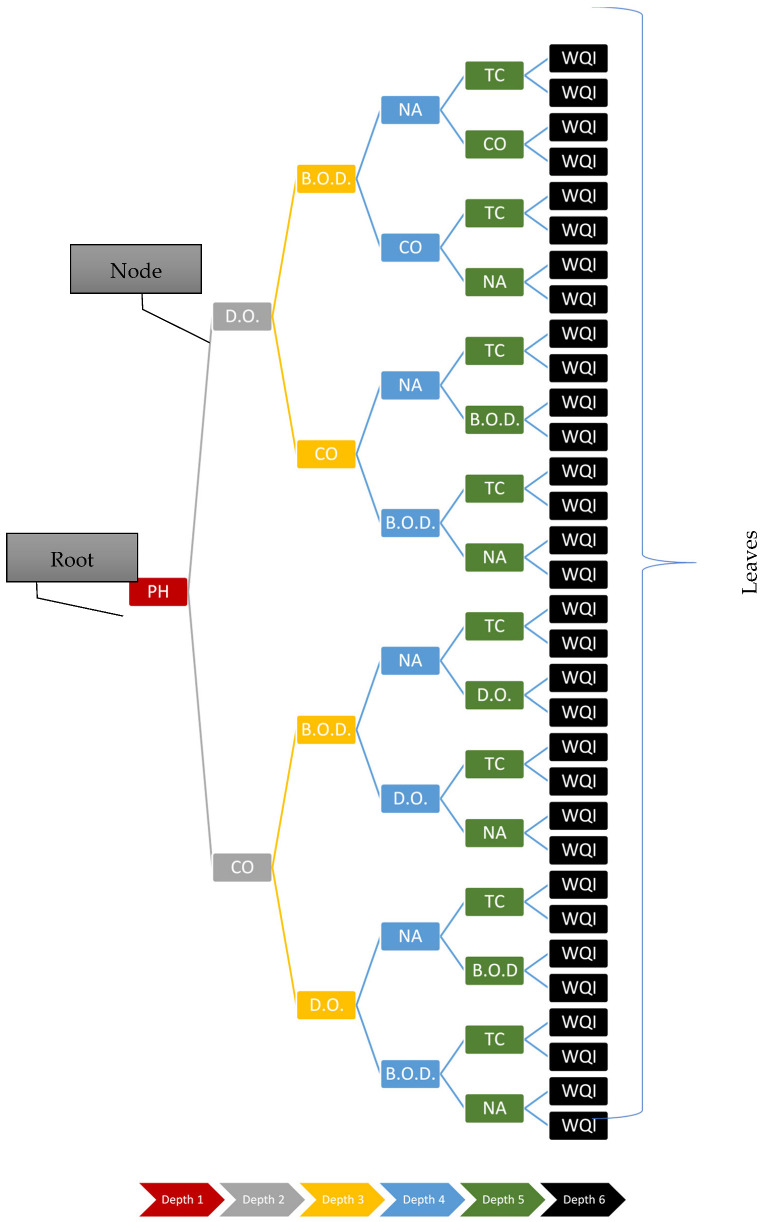
WQI decision tree.

A decision tree generated using the water quality data is represented in Figure 3. The ‘root’ represents the top-most decision node, a ‘node’ represents a decision node, and the leaves represent the final WQI predicted values, which is the final decision. 

The data split is achieved here using the fast divide and conquer greedy algorithm. However, this greedy algorithm might create bad decisions on deeper levels due to the instability of the estimations.

#### 3.3.2. Linear Regression

The LR algorithm links the independent variables $V_{i}$ to the dependent variable $V_{d}$ using Equation (5) [50].

(5) $$V_{d}=\beta_{0}+\beta_{1}V_{il}+\cdots +\beta_{n}V_{in}$$

The $\beta_{0}$ in the equation is the intercept value, and $\beta_{i}\;\left(i=1,\;2,\ldots ,\;n\right)$ are the coefficients of the descriptions/parameters. The $\beta_{i}$ values are obtained using the least square technique. $V_{i}$ refers to the parameters of the water quality {‘ph’, ‘do’, ‘co’, ‘bod’, ‘na’, ‘fc’}. Here, n = 6 is the number of parameters. In this study, there are three sets of parameters, and each set contains six descriptors, none of which overlap. 

#### 3.3.3. Ridge Regression 

Ridge regression is commonly used for data with independent and correlated variables. It overcomes the shortcoming of LR in dealing with highly correlated data using $\ell_{2}$ penalized least squares. The $\ell_{2}$ penalty avoids a sparse model. It is calculated using the square of coefficients magnitudes. The ridge regression coefficients are calculated using Equation (6) [51]:

(6) $${\hat{\beta }}_{Ridge}=\arg\min\;\|Y-X{\beta \|}_{2}^{2}+\lambda {\left\|\beta \right\|}_{2}^{2}$$

where λ>0 is the tuning parameter. The Y and X are the same as previously defined. 

#### 3.3.4. Lasso Regression

Lasso regression, which stands for Least Absolute Shrinkage and Selection Operator, is reported to work well with a large number of data, where systematic and rapid approaches are important, but it is not stable for highly correlated predictors [49]. The penalty calculation approach is expected to obtain a greater number of coefficients close to zero and a small number of coefficients with larger values. Lasso is also known as $\ell_{1}$ regularization, and the estimator definition is shown in Equation (7) [51]:

(7) $${\hat{\beta }}_{lasso}=\arg\min\;\|Y-X\beta {\|}_{2}^{2}+\lambda \|\beta {\|}_{1}$$

where *λ* ≥0 is the tuning parameter.

#### 3.3.5. Support Vector Regression

Here, the WQI value is also predicted using SVR and the water quality factors. The x space’s input vector is mapped onto a space with higher dimensions. This process is executed using the correct nonlinear kernel function, denoted as φ(x). To address this complex nonlinear regression of the input space, a simple linear regression is obtained. The SVR estimator $f_{SVM}$ is obtained using Equation (8) [9]:

(8) $$f_{SVM}=w\cdot \left(x\right)+b$$

where w represents the weight vector for the regression coefficient, while the value b indicates the biases of the estimator.

SVR has a good prediction performance reputation due to its enhanced optimization approaches that can be applied to a wide set of variables and kernels.

#### 3.3.6. Random Forest Regression

**Figure 4:**
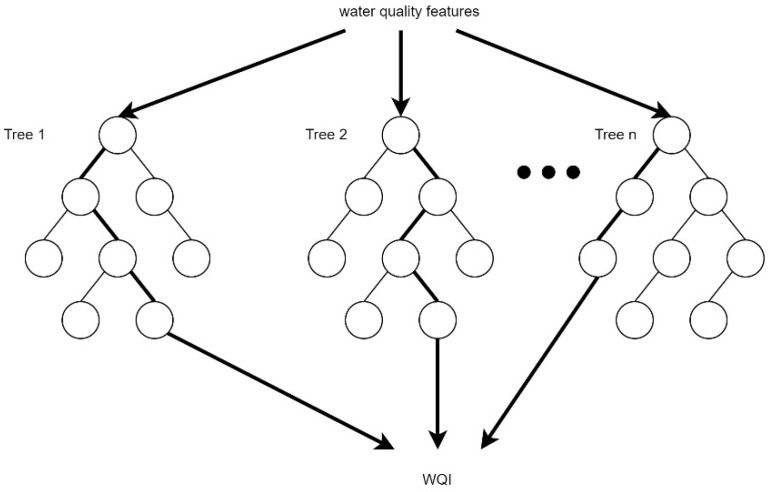
Flow of data in RF regression.

Similar to the DT algorithm, RF also generate trees. However, instead of one tree, it consists of multiple decision trees, which can be used to find the best tree with which to obtain the WQI value. The water quality factors are the features, and x and the WQI values are values which are factored into the model to create more than one decision tree, as shown in Figure 4.

This approach to predicting the WQI value helps us to obtain an unbiased estimate error of the generalization of the trees. The Gini impurity is used here to obtain the probability misclassification of each node. The best aspect of the algorithm is that it preserves a good regression accuracy even with very small and partially missing datasets. However, it may cause data overfitting and add noisy regression tasks. 

#### 3.3.7. Extra Tree Regression

ET is an extension of RF and an ensemble of DT. ET uses all the training set to train all the tress and makes its prediction by averaging the predictions from the decision trees. It is a highly randomized extension of RF and, thus, less prone to overfitting compared to RF. 

#### 3.3.8. ANN Regression

**Figure 5:**
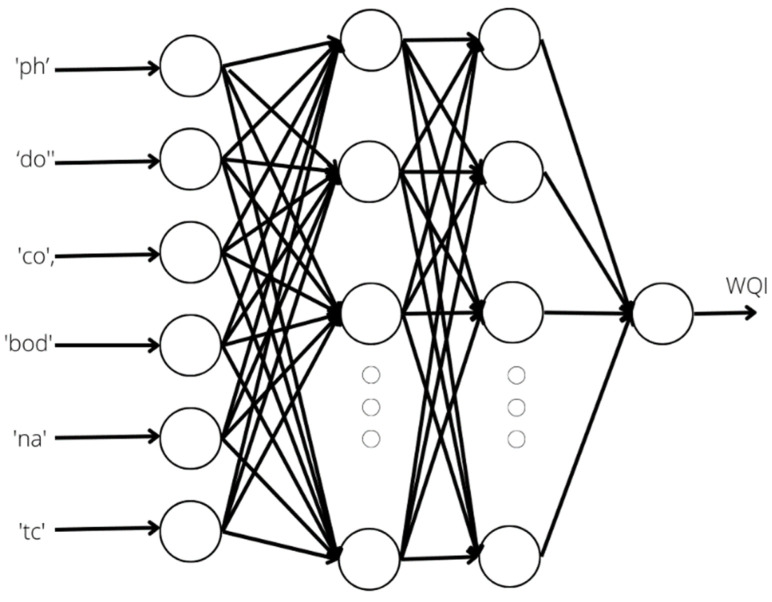
Architecture of the WQI artificial neural network regression (ANN).

An ANN is also used here for the WQI regression problem. The ANN is illustrated in Figure 5. A total of 6 inputs, including ‘ph’, ‘do’, ‘co’, ‘bod’, ‘na’, ‘tc’, as well as 2 hidden layers with 100 hidden neurons, are used to obtain the possible WQI value. An additional $x_{0}$ value, known as bias, is used as an extra weight, z (this weight is different from $w_{i}$), in each hidden layer. The biases help us to adjust the weighted sum of the output and input data for each neuron. The hidden layers are linked using weights, e.g., the neuron $z_{j}^{\left(i\right)}$ from the $i^{th}$ layer. The link is obtained using Equation (9):

(9) $$z_{j}^{\left(i\right)}=f\left(\overset{n}{\underset{k=1}{\sum }}x_{jk}^{\left(i-1\right)}\;z_{jn}^{\left(i-1\right)}\right)$$

where f() indicates the involvement of the activation function. In this work, the refined linear unit (reLU) function is used for all the hidden layers. However, for the output layer, the pure linear function is adopted. Here, n represents the number of neurons used in ${\left(i-1\right)}^{th}$ hidden layer.

### 3.4. System Evaluation

The statistical calculations of the mean square error (MSE), correlation coefficient (r), and mean absolute error (MAE) are utilized to measure the WQI prediction models’ performance. Additionally, the root mean square error (RMSE) is also measured, but only for the sake of comparison with the available works. The statistical values are calculated using Equations (10)–(13) [27,33]:

(10) $$MSE=\frac{1}{n}\overset{n}{\underset{i=1}{\sum }}{\left(y_{i}-{\hat{y}}_{i}\right)}^{2}$$

(11) $$r=\frac{\sum_{i=0}^{n}\left(x_{i}-\bar{x}\right)\left(y_{i}-\bar{y}\right)}{\sqrt{\sum_{i=0}^{n}{\left(x_{i}-\bar{x}\right)}^{2}{\left(y_{i}-\bar{y}\right)}^{2}}}$$

(12) $$MAE=\overset{n}{\underset{i=1}{\sum }}\left|y_{i}-{\hat{y}}_{i}\right|/n$$

(13) $$RMSE=\sqrt{MSE}$$

where n represents the overall number of data points, $y_{i}$ is the actual WQI value, and ${\hat{y}}_{i}$ is the predicted WQI value for the data point i. In the equation for the correlation coefficient, $x_{i}$ and $y_{i}$ are the values of the x-variable and y-variable, respectively, whereas $\bar{x}$ and $\bar{y}$ are the means of all the data points.

## 4. Results & Discussion

### 4.1. Regression Models Evaluation

The identification of the optimum regression model for WQI prediction from among the eight regression algorithms and three sets of input features is the main objective of this study. The data are divided into 80% training and 20% testing ratio groups. Table 4, Table 5 and Table 6 display the model prediction outcomes for the studied regression techniques and feature sets. The cells with the best results are shaded in grey. 

Based on the MSE, it can be observed that all the regression algorithms using feature set 2, $qi_{2}\;$= (‘npH’, ‘ndo’, ‘nbdo’, ‘nec’, ‘nna’, ‘nco’), performed better in comparison to the models built using the same regression algorithms trained with set 1. Among the models trained using $qi_{2}$, the LR model and Ridge have the lowest MSE, which is equal to 0. The correlation coefficient values in Table 5 also show that LR and Ridge have the highest correlation coefficients, which are equal to 1. It is also observed that $qi_{2}$ is the best input for LR, Ridge, Lasso, and ANN. Meanwhile, $qi_{3}$ is the best input for DT, SVR, RF and ET. Furthermore, the calculated MAE results of LR and Ridge also have very low values, which are 1.$3843\times 10^{-14}$ and $1.2872\times 10^{-5}$, respectively. Set 2, $qi_{2}$= [‘wph’, ‘wdo’, ‘wco’, ‘wbod’, ‘wna’, ‘wfc’], is also found to produce the lowest MAE for all the algorithms, with the exception of DT.

**Figure 6:**
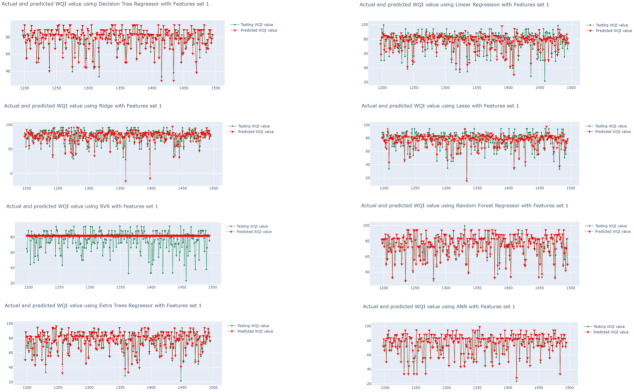
Time series visualization of actual and predicted WQI values (WQI vs. time) using set 1, $qi_{1}$.

**Figure 7:**
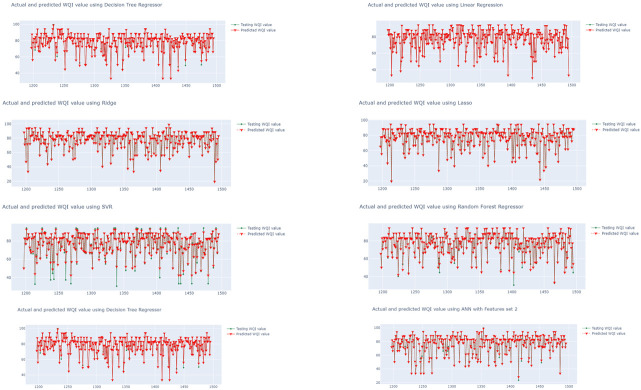
Time series visualization of actual and predicted WQI values (WQI vs. time) using set 2, $qi_{2}$.

**Figure 8:**
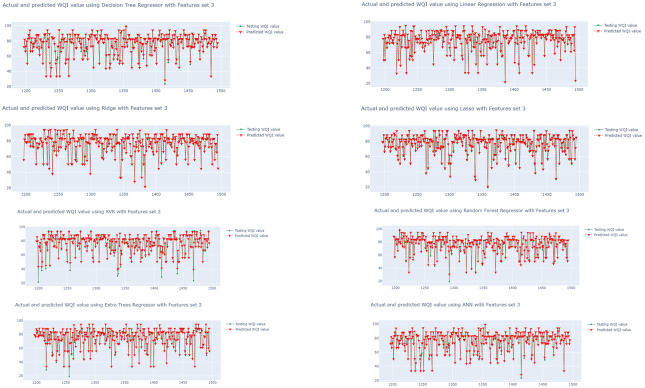
Time series visualization of actual and predicted WQI values (WQI vs. time) using set 3, $qi_{3}$.

The performances of the eight algorithms are illustrated in Figure 6, Figure 7 and Figure 8. The *y*-axis in the graphs is the WQI value and the *x*-axis is the time. The graphs in Figure 6 show the outputs of the models trained using the eight regression algorithms and feature set 1 (i.e., $\;qi_{1}$), while Figure 7 and Figure 8 presents the predicted outputs for feature set 2 (i.e., $\;qi_{2}$) and feature set 3 (i.e., $\;qi_{3}$), respectively. The visualization in Figure 7 shows that the LR- and Ridge-trained models provided almost exact plot predictions, where the predicted WQI (red) overlapped with the actual/testing WQI (green) value. The worst predictive model is that trained with the combination of SVR and$\;qi_{1}$ (Figure 6), and it can be seen that there is almost no overlap between the predicted and actual values. Additionally, the MSE is as high as 191.9587, and the correlation coefficient observed is 0.4457, while the MAE is 9.31485. From the three figures, it can be seen that the regression models trained using $qi_{2}$ are better, with more overlaps between the actual and predicted values for all of the eight models. In contrast, the models trained using raw data ($qi_{1}$) have poor performances, with a greater number of inaccurate predictions. The regression models using the water quality weight score, $qi_{3}$, have a better performance than those using $qi_{1}$ and are almost as effective as those using $qi_{2}$.

Overall, it is observed that the regression algorithms influence the WQI prediction system’s performance. Additionally, one of the most critical performance influencers is the set of the features used to train the models. The raw features tend to perform the worst, whereas the derivative features of the water quality rating and weight score ($qi_{2},\;qi_{3})$ contribute to a better performance. With these derivative features, the LR and Ridge are the more robust and flexible standalone models, with the lowest prediction error and highest correlation. 

### 4.2. ANN Models Evaluation

From Section 2, it can be seen that ANN is the popular choice among researchers in this field. Therefore, the ANN models trained using different features (i.e., $qi_{1},\;qi_{2},\;qi_{3}$) are closely analyzed here. 

**Figure 9:**
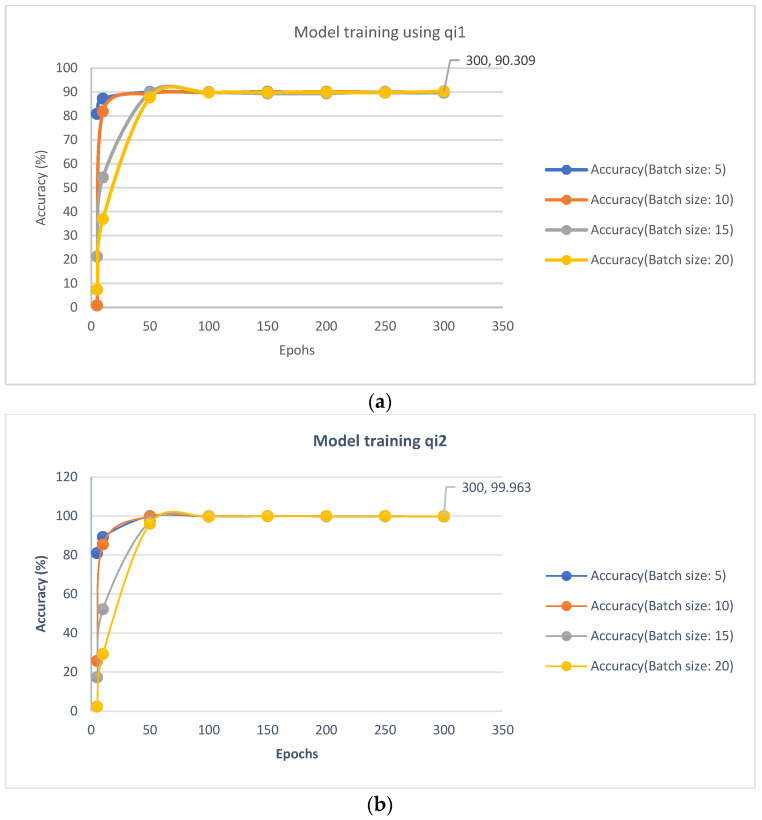

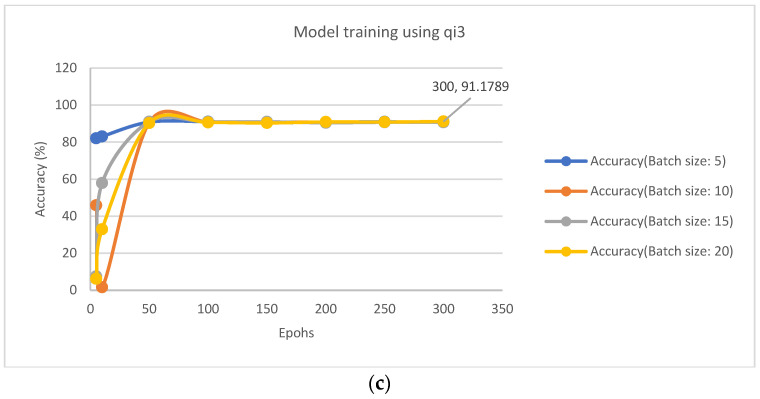
Training ANN models using water quality parameters (**a**)$\;qi_{1}$, (**b**)$\;qi_{2}$, and (**c**) $\;qi_{3}$.

The accuracy of the model is presented in Figure 9. It can be observed that$\;qi_{2}$ provides a better prediction model, where the accuracy is 99.963%, whereas the set$\;qi_{1}$ gives an accuracy of 90.309% and set$\;qi_{3}$ has an accuracy of 91.1789%, which is slightly better than that of$\;qi_{1}.$ It is also observed that, with a larger batch size, the accuracy increases more gradually compared to a smaller batch size. This is to be expected, and the finding follows the trend usually observed for ANN.

### 4.3. Comparison with Existing Works

The performances of the recent and closely related models and the models obtained in this work are compared in Table 7. The existing works used the ET, ANN, SVM, least square SVM (LS-SVM), BA-RT, long short-term memory (LSTM), and MLP. Some of the parameters adopted are the same as those available in the dataset used in this work. All the works reported the correlation value, but not all reported the RMSE or MSE values.

One work [44] used the same dataset as the one adopted here. It can be seen that the proposed work reported the best correlation value and RMSE and MSE values, which are 1, 0, and 0, respectively. These are also better than the findings of [44]. The better results are contributed by the regression algorithm, as well as the features used.

## 5. Future Works and Challenges

The findings suggest that LR and Ridge are the best regression algorithms for water quality prediction systems, while the water quality rating scale is the best input for the model. As can be seen from previous research [27,28,29], not all the features are important, and selecting the best combination leads to a better prediction model. Hence, in the future, a more in-depth study on the effect of the feature combination and its importance should be pursued.

In a paper issued by the International Telecommunication Union (ITU) [53], AI’s ability to support the UN’s SDG is acknowledged. However, despite the many benefits of AI-based prediction systems, such as their capacity for automated data pattern and trend analysis [54], ability to predict complex, nonlinear systems [18,55], and capacity to handle noisy and large dynamic data [55], the adoption of AI systems in environment science, including water quality and hydrology studies, faces many challenges. Among the main challenges, as listed in [56,57], are the following: (1) The heavy usage of historical data for machine learning training causes biased models, as the modelled systems are frequently dynamic systems. For example, the water quality is affected by climate, which is highly dynamic. (2) In comparison to the ways in which humans make decisions and form responses, AI-based systems are considered static and less adaptive. (3) An AI-based system is also prone to cyber security issues. (4) Moreover, training a machine learning system is a costly process that leaves a large carbon footprint, and (5) stakeholders, such as policy makers and communities, need to be convinced of, trained in, and educated on the application of AI. Additionally, the digital divide between the world populations is another challenge for AI system adoption [53]. Economically, AI is expected to have a positive impact [53]. However, these challenges need to be addressed so that the benefits can be fully gained. 

## 6. Conclusions

AI solutions such as machine learning ease the task of WQI prediction. The AI-based WQI prediction system supports efforts to provide timely and efficient water pollution prevention and response systems by forecasting the change in the WQI based on historical data. In this paper, eight standalone machine learning regression algorithms (DT, LR, Ridge, Lasso, SVR, RF, ET and ANN) were compared for their predictions of the WQI using three sets of water parameter features. An open dataset based on data from Indian rivers collected between 2003 to 2014 was used. The WQI was measured using six water quality features, including the pH, DO, CO, BOD, NA, and FC. Two sets of derivative features were derived, namely the water quality rating scale and water quality weight score. The original water quality features and the two sets of derivative features were then used in the WQI prediction. The results show that LR and Ridge trained using the water quality rating scale are able to predict the WQI accurately, with MSE=0 and r=1. The results outperformed the performances of existing models. Overall, it was observed that the regression algorithm and set of features used are the main factors affecting the performance of an WQI prediction model. Future research directions and challenges were also addressed in this work.

## Figures and Tables

**Table 1:** Existing works.

Ref.	Data Origin	Prediction Algorithm	Parameters
[9]	Lam Tsuen River, Hong Kong	ET (vs. SVR & DT)	BOD, COD, DO, CO, pH, NO_3−_N, NO_2−_N, PO_4_^3−^, T, and TUR
[27]	Talar, Iran	M5P; RF; random tree (RT); reduced error pruning tree (REPT); BA-M5P; BA-RF; BA-RT; BA-REPT; CVPS-M5P; CVPS-RF; CVPS-RT; CVPS-REPT; RFC-M5P; RFC-RF; RFC-RT; RFC-REPT (where; bagging (BA); CV parameter selection (CVPS); and randomizable filtered classifier (RFC))	BOD, COD, DO, pH, TS, FC, PO_4_^3−^, NO_3−_, TUR, and CO (FS: the most important, TS: the least important)
[28]	Syczyn, Lublin Province, Poland	ANN	CO, pH, Ca, Mg, PO_4−_P, K, and SO_4_^2−^ (best set: CO, pH, Ca, Mg, K)
[29]	Klang, Malaysia	ANN	COD, BOD, DO, SS, pH, and AN (DO: the most important, pH: the least important)
[30]	Langat, Malaysia	ANN	31 parameters
[31]	Klang and Langat, Malaysia	ANN (BPNN & RBFNN)	DO, BOD, COD, SS, AN, and pH
[32]	Lake Qaroun, Egypt	ANN	TN, NH_4_^+^, PO_4_^3−^, and COD
[33]	Tyhume River, Bloukrans River, Buffalo River, Eastern Cape Province, South Africa	ANN (MLP)	Input: T, chloride, sulfate, and PO_4_^3−^ Output: pH, CO, DO, and TUR
[34]	Warta River, Poland	ANN	TDS, chloride, TH, NO^3−^, and manganese
[35]	120 rivers and lakes, China	Multi-task learning and deep neural network (vs. LR, XGBoostmodel, MLP, CNN, LSTM, GRU, and ATTENTION)	pH, DO, COD, and AN

**Table 2:** Parameter unit weights$\;w_{i}$ and recommended standard values of the parameters used for calculating the WQI.

Water Quality Parameters	$w_{i}$	$Ideal_{i}$	$Standard_{i}$
Dissolved oxygen	0.281	14.6 mg/L	10 mg/L
pH	0.165	7	8.5
Conductivity	0.281	0 μS/cm	1000 μS/cm
Biological oxygen demand	0.234	0 mg/L	5 mg/L
Nitrate	0.028	0 mg/L	45 mg/L
Fecal coliform	0.281	0 Cfu/100 mL	100 Cfu/100 mL

**Table 3:** Different input feature combinations.

Set Number	Feature Combination
1	$qi_{1\;}$= [‘ph’, ‘do’, ‘co’, ‘bod’, ‘na’, ‘fc’]
2	$qi_{2}\;$= [‘npH’, ‘ndo’, ‘nco’, ‘nbod’, ‘nna’, ‘nfc’,]
3	$qi_{3\;}$= [‘wph’, ‘wdo’, ‘wco’, ‘wbod’, ‘wna’, ‘wfc’]

**Table 4:** Models’ mean square errors, MSE.

WQI WEIGHT	DT	LR	Ridge	Lasso	SVR	RF	ET	ANN
$qi_{1}$	8.2011	62.1054	60.6084	57.3244	191.9587	15.6543	7.9947	90.6694
$qi_{2}$	1.9124	0	0	0.0071	2.7043	1.7122	1.5602	0.1415
$qi_{3}$	1.0522	0	0.0025	0.3230	2.5803	0.9258	1.4879	1.3240

**Table 5:** Models’ correlation coefficients, r.

WQI WEIGHT	DT	LR	Ridge	Lasso	SVR	RF	ET	ANN
$qi_{1}$	0.9781	0.7912	0.7841	0.8133	0.4457	0.9459	0.9772	0.7575
$qi_{2}$	0.9933	1	1	0.9999	0.9917	0.9942	0.9947	0.9995
$qi_{3}$	0.9965	1	0.9999	0.9995	0.9953	0.9975	0.9966	0.9966

**Table 6:** Models’ mean absolute errors, MAE.

WQI WEIGHT	DT	LR	Ridge	Lasso	SVR	RF	ET	ANN
$qi_{1}$	0.9465	5.8896	6.0249	5.9968	9.3149	2.1533	1.6170	4.7167
$qi_{2}$	0.2457	1.3843$\times 10^{-14}$	1.2872$\times 10^{-5}$	0.0677	0.5926	0.2348	0.1867	0.1137
$qi_{3}$	0.17458	1.9879$\times 10^{-14}$	0.0052	0.4633	0.6988	0.2649	0.2274	0.2193

**Table 7:** Comparing the proposed models to recent existing models.

Reference & Source	Model Used	Parameters	Predicted Value(s)	r	*RMSE*	MSE
[9] Lam Tsuen River, Hong Kong	ET	BOD, COD, DO, EC, pH, NO_3_-N, NO_2_-N, PO_43_-, T, and TUR	WQI	0.98	2.99	
		BOD, TUR, PO_43_-	WQI	0.97	3.74	
[28] Syczyn, Lublin Province, Poland	ANN	EC, pH, Ca, Mg, K	WQI	0.9992		0.2131
[52] Perak, Malaysia	SVM	COD, BOD, DO, AN, SS, pH	WQI	0.9184		
	LS-SVM		WQI	0.9227		
[29] Klang, Malaysia	ANN	COD, BOD, DO, AN, SS, pH	WQI	98.78		
[27] Talar, Iran	BA-RT	BOD, COD, DO, pH, TS, FC, PO_43_-, NO_3_-, TUR, and EC	WQI	0.941	2.71	
[44] India	ANN	pH, DO, CO, BOD, NA, FC	WQI	0.9617		
	LSTM		WQI	0.9421		
[32] Lake Qaroun, Egypt	ANN	TN, NH_4_^+^, PO_43_^−^, and COD	PO_43_^−^	0.98		
[33] Eastern Cape Province, South Africa	ANN(MLP)	pH, EC, DO, and TUR	pH, EC, DO, TUR	0.9935		39.0308
[34] Warta River, Poland	ANN	TDS, chloride, TH, NO_3_-, and manganese	WQI	0.9792	0.62450	
Proposed model, India	LR, Ridge	pH, DO, CO, BOD, NA, FC	WQI	1	0	0

## Data Availability

The dataset used in this study was obtained from Kaggle, https://www.kaggle.com/datasets/anbarivan/indian-water-quality-data (accessed on 1 December 2021).

## List of Abbreviations

**Abbreviation**	**Water Parameter**	**Abbreviation**	**Machine Learning Algorithm**	**Abbreviation**	**Others**
AN	ammoniacal nitrogen	ANN	artificial neural network	$Actual_{i}$	actual value of parameter i in the tested water samples
BOD	biological oxygen demand	BPNN	back propagation NN	AI	artificial intelligence
Ca	calcium	DT	decision tree regression	$Ideal_{i}$	the optimal parameter value i in pure water
CO	electrical conductivity	ET	extra tree regression	MAE	mean absolute error
COD	chemical oxygen demand	LR	linear regression	MSE	mean square error
DO	dissolved oxygen	MLP	multilayer perceptron	r	correlation coefficient
FC	faecal coliform	RBFNN	radial basis function NN	RMSE	root mean square error
K	potassium	RF	random forest regression	SDG	Sustainable Development Goals
Mg	magnesium	SVR	support vector regression	$Standard_{i}$	the suggested parameter standard value i
NH_4_^+^	ammonium			UN	United Nation
NO_3−_ i.e., Na	nitrate			UNEP	United Nations Environment Program
NO_3−_N	nitrate-nitrogen			$w_{i}$	each feature’s unit weight
NO_2−_N	nitrite-nitrogen			WQI	Water Quality Index
pH	potential for hydrogen			$WQR_{i}$	Water quality rating scale for each feature i
PO_4_^3-^	Phosphate			$WQWS_{i}$	water quality weight score for each feature i
PO_4−_P	phosphate				
SO_4_^2−^	sulfur				
SS	suspended solids				
T	temperature				
TC	total coliform				
TDS	total dissolved solids				
TH	total hardness				
TN	total nitrogen				
TS	total solids				
TUR	turbidity				
